# Sister *Dehalobacter* Genomes Reveal Specialization in Organohalide Respiration and Recent Strain Differentiation Likely Driven by Chlorinated Substrates

**DOI:** 10.3389/fmicb.2016.00100

**Published:** 2016-02-12

**Authors:** Shuiquan Tang, Po Hsiang Wang, Steven A. Higgins, Frank E. Löffler, Elizabeth A. Edwards

**Affiliations:** ^1^Department of Chemical Engineering and Applied Chemistry, University of TorontoToronto, ON, Canada; ^2^Department of Microbiology, University of TennesseeKnoxville, TN, USA; ^3^Center for Environmental Biotechnology, University of TennesseeKnoxville, TN, USA; ^4^University of Tennessee and Oak Ridge National Laboratory Joint Institute for Biological Sciences and Biosciences Division, Oak Ridge National LaboratoryOak Ridge, TN, USA; ^5^Department of Civil and Environmental Engineering, University of TennesseeKnoxville, TN, USA

**Keywords:** *Dehalobacter*, organohalide respiration, genome analysis, reductive dehalogenase, microbial evolution

## Abstract

The genomes of two closely related *Dehalobacter* strains (strain CF and strain DCA) were assembled from the metagenome of an anaerobic enrichment culture that reductively dechlorinates chloroform (CF), 1,1,1-trichloroethane (1,1,1-TCA) and 1,1-dichloroethane (1,1-DCA). The 3.1 Mbp genomes of strain CF (that dechlorinates CF and 1,1,1-TCA) and strain DCA (that dechlorinates 1,1-DCA) each contain 17 putative reductive dehalogenase homologous (*rdh*) genes. These two genomes were systematically compared to three other available organohalide-respiring *Dehalobacter* genomes (*Dehalobacter restrictus* strain PER-K23, *Dehalobacter* sp. strain E1 and *Dehalobacter* sp. strain UNSWDHB), and to the genomes of *Dehalococcoides mccartyi* strain 195 and *Desulfitobacterium hafniense* strain Y51. This analysis compared 42 different metabolic and physiological categories. The genomes of strains CF and DCA share 90% overall average nucleotide identity and >99.8% identity over a 2.9 Mbp alignment that excludes large insertions, indicating that these genomes differentiated from a close common ancestor. This differentiation was likely driven by selection pressures around two orthologous reductive dehalogenase genes, *cfrA* and *dcrA*, that code for the enzymes that reduce CF or 1,1,1-TCA and 1,1-DCA. The many reductive dehalogenase genes found in the five *Dehalobacter* genomes cluster into two small conserved regions and were often associated with Crp/Fnr transcriptional regulators. Specialization is on-going on a strain-specific basis, as some strains but not others have lost essential genes in the Wood-Ljungdahl (strain E1) and corrinoid biosynthesis pathways (strains E1 and PER-K23). The gene encoding phosphoserine phosphatase, which catalyzes the last step of serine biosynthesis, is missing from all five *Dehalobacter* genomes, yet *D. restrictus* can grow without serine, suggesting an alternative or unrecognized biosynthesis route exists. In contrast to *D. mccartyi*, a complete heme biosynthesis pathway is present in the five *Dehalobacter* genomes. This pathway corresponds to a newly described alternative heme biosynthesis route first identified in Archaea. This analysis of organohalide-respiring *Firmicutes* and *Chloroflexi* reveals profound evolutionary differences despite very similar niche-specific metabolism and function.

## Introduction

Chlorinated hydrocarbons, including chlorinated ethenes, ethanes, and methanes are common groundwater contaminants (De Wildeman and Verstraete, 2003; Löffler and Edwards, 2006). A viable approach for groundwater detoxification is via bioremediation using microbes capable of organohalide respiration (Major et al., 2002; Lendvay et al., 2003; Ward and Stroo, 2010). Organohalide-respiring bacteria are phylogenetically diverse and include members of the *Chloroflexi, Firmicutes*, and *Proteobacteria* (Löffler and Edwards, 2006; Maphosa et al., 2010; Hug et al., 2013). *Dehalobacter* spp. dechlorinate chlorinated ethenes (Wild et al., 1996; Holliger et al., 1998), chlorinated ethanes (Sun et al., 2002; Grostern and Edwards, 2006a,b; Grostern et al., 2010), chlorobenzenes (Nelson et al., 2011), and other halogenated compounds (Schlotelburg et al., 2002; van Doesburg et al., 2005; Yoshida et al., 2009a,b; Wang et al., 2014). The genus *Dehalobacter* belongs to the phylum *Firmicutes* and is phylogenetically closely related to the genus *Desulfitobacterium* (Villemur et al., 2006). But unlike *Desulfitobacterium* spp., which are known for their metabolic versatility, most members of the *Dehalobacter* group are specialized for organohalide respiration, a lifestyle akin to *Dehalococcoides* or *Dehalogenimonas* in the *Choroflexi* (Siddaramappa et al., 2012). However, two *Dehalobacter* populations capable of fermentation of dichloromethane were recently described (Justicia-Leon et al., 2012; Lee et al., 2012), indicating members of the *Dehalobacter* genus can be more versatile than *Dehalococcoides*.

Previously, we reported the *de novo* assembly of the first two complete *Dehalobacter* genomes from metagenomic data derived from an enrichment culture called ACT-3, that dechlorinates 1,1,1-trichloroethane (1,1,1-TCA) to chloroethane (CA) via 1,1-dichloroethane (1,1-DCA), and also dechlorinates chloroform (CF) to dichloromethane (DCM; Tang et al., 2012). In this culture, the two-step dechlorination of 1,1,1-TCA to 1,1-DCA followed by 1,1-DCA to CA is catalyzed by two distinct *Dehalobacter* populations. The first population dechlorinates 1,1,1-TCA to 1,1-DCA and CF to DCM using a specific reductive dehalogenase (RDase), CfrA, while the second population dechlorinates 1,1-DCA to CA using a distinct RDase, DcrA. We have now isolated strain CF and strain DCA corresponding to the two assembled genomes (Tang, 2014). We also previously found that the two active RDases CfrA and DcrA belonging to each strain share 95.2% amino acid identity and 97.8% nucleotide identity, although one dechlorinates CF and 1,1,1-TCA and the other dechlorinates 1,1-DCA, with no cross-reactivity. These were the only two RDase genes found expressed in the ACT- 3 culture grown with 1,1,1-TCA as electron acceptor (Tang and Edwards, 2013).

In this paper, we report the detailed analysis of these two *Dehalobacter* genomes in comparison with three other *Dehalobacter* genomes: the complete genome of *Dehalobacter restrictus* strain PER-K23 (Kruse et al., 2013), the draft genome of *Dehalobacter* sp. strain E1 (Maphosa et al., 2012) and the draft genome of *Dehalobacter* sp. strain UNSWDHB (Deshpande et al., 2013). Additionally, we compare *Dehalobacter* genomes to those of *Desulfitobacterium hafniense* Y51 (Nonaka et al., 2006) and *Dehalococcoides mccartyi* strain 195 (Seshadri et al., 2005) as contrasting organohalide respiring bacteria. The physiological characterization of strains CF and DCA is on-going and the complementary experimental data in Tang's PhD thesis (Tang, 2014) will be reported separately.

## Results and discussion

### General genome features

The three complete *Dehalobacter* genomes of strains CF, DCA and PER-K23 are similar in size (~3.0 Mb) and G+C content (44–45%) and comprise ~2900 coding sequences (CDS; Table 1). The draft genome of strain E1 was sequenced with 454 pyrosequencing with ~13 × coverage and consists of 102 contigs (Maphosa et al., 2012). The draft genome of strain UNSWDHB was Illumina paired-end sequenced with 230 × coverage and consists of 220 contigs (Deshpande et al., 2013). Because the draft genomes of strains E1 and UNSWDHB were assembled from short-read sequencing data, long interspersed repeats, such as multi-copy transposable elements (TEs) and ribosomal RNA (rRNA) genes, are not well represented in these two draft genomes.

**Table 1:** **General features of the five *Dehalobacter* genomes**.

	**Strain CF**	**Strain DCA**	**Strain PER-K23**	**Strain E1**	**Strain UNSWDHB**
Genome size (Mbp)	3.09	3.06	2.9	2.6^a^	3.2
G+C content (%)	44.3	44.6	44.6	43.8	44.9
Protein coding genes	2980	2978	2826	2587^a^	2489
rRNA operon	3	3	4	N/A	N/A
tRNA	51	51	52	55^a^	N/A
Genes with function prediction	2072	2014	2168	N/A	N/A
Genes with COGs	2167	2174	2127	N/A	N/A
Genes with KEGG pathways	749	751	740	N/A	N/A
Genes encoding transmembrane proteins	724	739	755	N/A	N/A
Insertion sequences	68	71	69	N/A	N/A
*rdhA* genes	17	17	25	10^a^	17
Chlorinated substrates	1,1,1-TCA, CF	1,1-DCA	PCE	β-HCH	CF
Active (i.e., expressed) *rdhA* genes (locus_tag)	*cfrA* (DCF50_p1247)	*dcrA* (DHDCA_p1180)	*pceA* (Dehre_2398)	N/A	N/A
Main RdhA ID	AFV05253	AFV02209	CAD28790	N/A	N/A
NCBI genome accession number	CP003870	CP003869	CP007033	CANE00000000	AUUR00000000
Location of origin	US	US	Netherlands	Netherlands	Australia

a *Data were adapted from a previous report (Maphosa et al., 2012)*.

*“N/A” indicates not available*.

Overall, these five genomes share similar features (Table 1), consistent with the overall similarity of their 16S rRNA genes. Some 16S rRNA genes in strains CF, DCA, and UNSWDHB include an insertion near the 5′ end (Figure S1). Sequence insertions were also found in some 23S rRNA genes in the three complete *Dehalobacter* genomes (data not shown). Sequence insertions within 16S rRNA genes have been reported in some *Desulfitobacterium* strains (Villemur et al., 2007) but their meaning is unknown. The 16S rRNA gene sequences of strains CF, DCA and UNSWDHB (excluding insertions) are identical and differ from those of strains PER-K23 and E1 by only 10 nucleotides. In other words, the 16S rRNA genes for all five strains share >99% nucleotide identity. To better distinguish the five strains, a phylogenetic tree based on an alignment of concatenated orthologous genomic regions was constructed (Figure S2). The greater resolution offered by this tree separates strains CF, DCA, and UNSWDHB from the other two strains, and confirms that strains CF and DCA are more similar to each other than to any of the other strains.

**Figure 1:**
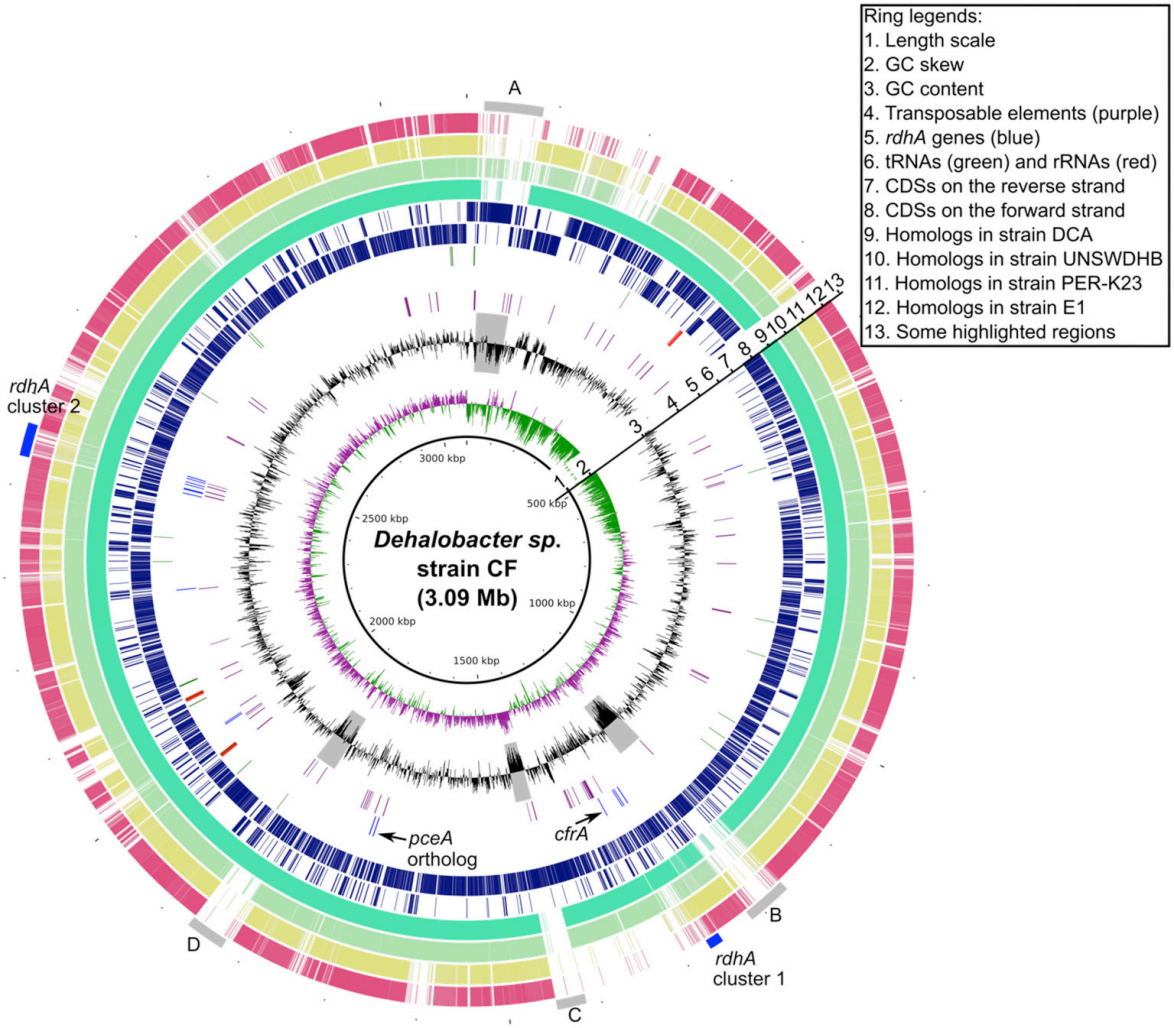
**Circular Genome map of *Dehalobacter* sp. strain CF outlining major features and relationship to strains DCA, UNSW, PER-K23, and E1**. The gray bars (labeled A, B, C, and D, ring 13) highlight some variable regions and the two blue bars, ring 13, hightlight the two *rdhA* clusters.

The DNA replication origin of the genomes of strains CF and DCA (Figure 1) was predicted based on the transition of GC-skew and the presence of a *dnaA* gene. A distinctive feature of these two genomes is the presence of two GC-skew arms of significantly different size. In contrast, the genome of strain PER-K23 has two GC-skew arms of equal size (Kruse et al., 2013). A similar phenomenon was seen in two *Desulfitobacterium* genomes and is believed to relate to genome rearrangement events (Nonaka et al., 2006; Kim et al., 2012). We have found that the difference in GC-skew profile and overall gene synteny between strains CF and PER-K23 can be accounted for by three potential genome rearrangements, which are described in more detail below.

### Recent differentiation between strains CF and DCA

**Figure 2:**
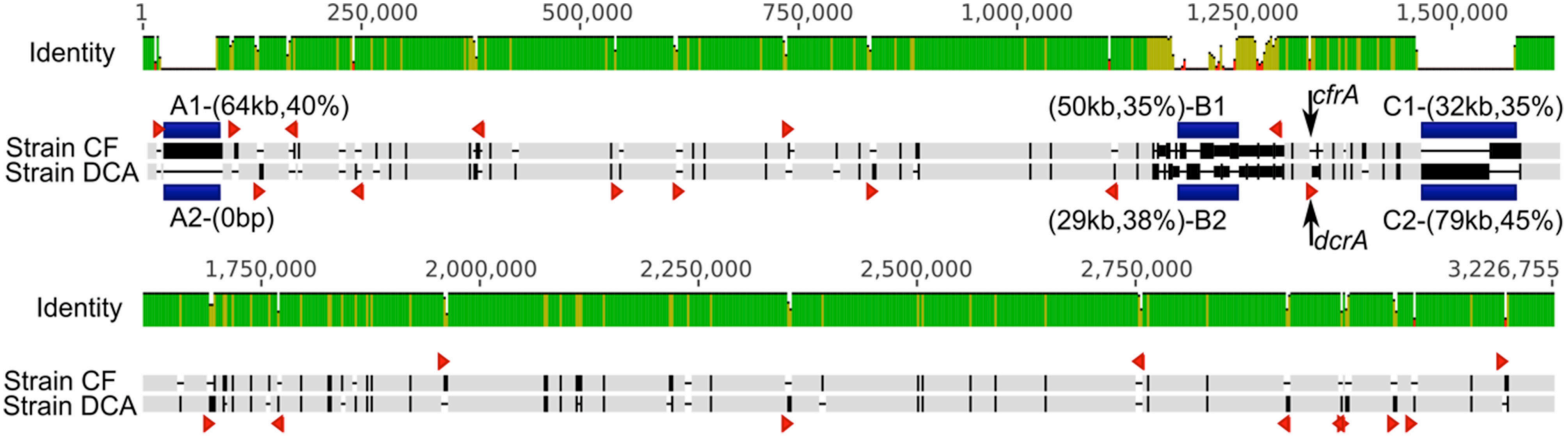
**Whole genome alignment of strains CF and DCA**. Sequence discrepancies are highlighted in black (compared to light gray) in the aligned sequences; this is a feature common to all sequence alignments presented in this paper. Blue blocks highlight three major regions of sequence variations: A1/A2, B1/B2, and C1/C2, with size and G+C content in parentheses. Red triangles represent strain-specific insertion sequences. Two arrows show the loci of *cfrA* and *dcrA*. The two genomes have a nucleotide identity of ~90% in this alignment.

**Figure 3:**
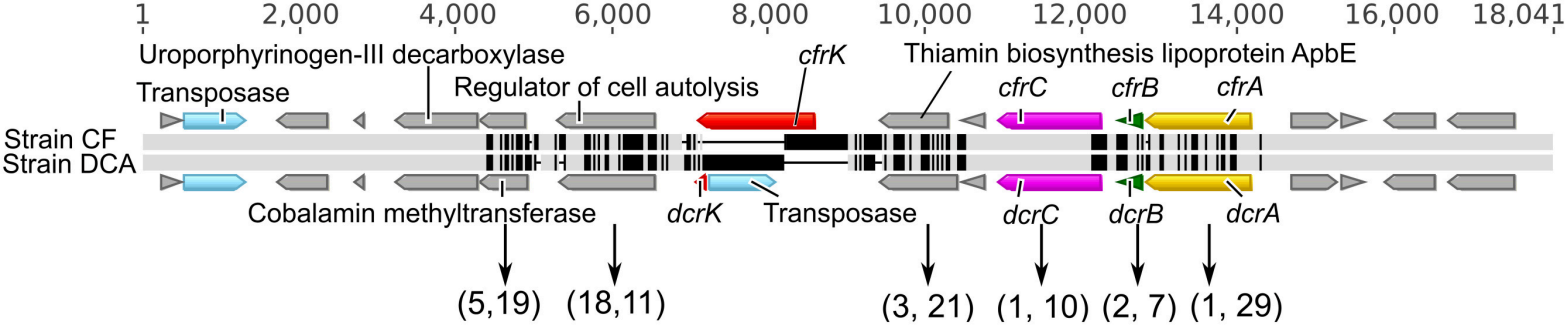
**Sequence alignments of the *cfrA* and *dcrA* gene neighborhoods**. The alignment is represented by two horizontal bars where light gray means that the residue at the position is the same in both sequences while black indicates substitutions. All coding sequences (CDSs) are indicated as directional blocks in different colors: *rdhA* genes (yellow), *rdhB* genes (green), *pceC*-like genes (purple), crp/fnr transcriptional regulators (red), ISs (light blue) and others (gray). The pairs of numbers in parentheses below are the counts of synonymous and non-synonymous substitutions (total 30 and 97, respectively).

The genomes of strain CF (3.09 Mb) and strain DCA (3.06 Mb) are remarkably similar to each other, sharing 90% average nucleotide identity based on a Mauve (Darling et al., 2010) alignment (Figure 2). After removing large insertions (genomic islands and strain-specific transpositions) and non-orthologous regions, the resulting alignment of 2,921,361 bp had an identity of 99.87%. No genome rearrangements were found between these two genomes. They share 2753 orthologous genes and most of these orthologous genes are identical. Conserved gene synteny and high sequence similarity suggest that strains CF and DCA have differentiated recently. The genome of strain CF contains 17 intact reductive dehalogenase homologous subunit A genes (*rdhA*) and only two differ from their orthologs in strain DCA. The first, DCF50_p1199, differs from its ortholog in strain DCA, DHBDCA_p1132, by 25 nucleotides out of 1542 corresponding to six amino acids and is not closely related to any other *rdhA* gene of known function. These two orthologous genes were not found expressed in the ACT-3 culture (Tang and Edwards, 2013). The second, *cfrA* (DCF50_p1247), is the gene encoding the RDase CfrA. Its ortholog in strain DCA is the *dcrA* gene (DHBDCA_p1180) encoding DcrA. CfrA and DcrA differ by 22 amino acids. The gene *cfrA* resides in a cluster (Figure 3) that includes the membrane anchor *cfrB* as well as *cfrC* (*pceC*-like), and *cfrK* (a crp/fnr transcriptional regulator) (Häggblom and Bossert, 2003). Between *cfrC* and *cfrK*, there is a gene (DCF50_p1243) annotated as thiamin biosynthesis lipoprotein ApbE, which might be involved in the maturation of iron-sulfur clusters (Skovran and Downs, 2003). The characterized RDases have two iron-sulfur clusters presumably involved in electron transfer to the cobalt atom of the corrinoid cofactor. In strain DCA, *dcrA* resides in a gene neighborhood orthologous to that of *cfrA* (Figure 3). More sequence variation between the two strains was found in the *cfrA* and *dcrA* gene neighborhoods compared to the rest of the genomes (Figure 3). Most of the variation exists in single nucleotide polymorphisms (SNPs) except for a transposase insertion (DHBDCA_p1176) in the *dcrA* gene cluster that disrupts the crp/fnr transcriptional regulator (Figure 3).

Sixty-seven (67) of the 70 SNPs that distinguish strains CF and DCA in the region between *cfrA* and *cfrK* are non-synonymous substitutions causing amino acid changes (Figure 3). This is a unique feature of this region, not observed elsewhere in these genomes. For example, only 7 of 25 SNPs between *rdh* sequence DCF50_p1199 and its ortholog in strain DCA are non-synonymous. These observations indicate that the *cfrA* and *dcrA* gene neighborhoods differentiated relatively recently and that the differentiation is under strong positive selection pressure. The strong positive selection in the *cfrA*/*dcrA* neighborhood signifies that their dechlorinating functions directly affect the fitness of their hosts. These sequence variations have caused the two strains to become specialized (one on CF and 1,1,1-TCA, and the other on 1,1-DCA), thus the positive selection appears driven by the presence of these chlorinated substrates. Since 1,1,1-TCA was the original contaminant at the site, and 1,1-DCA is not known to be naturally occurring, the original strain likely dechlorinated at least 1,1,1-TCA or perhaps both 1,1,1-TCA and 1,1-DCA, although other scenarios are possible, including evolution during laboratory enrichment.

Chloroform is naturally occurring in soils and other environments (Laturnus et al., 2002) while 1,1,1-TCA and 1,1-DCA have not been reported as natural organohalides. In the two other organisms capable of chloroform dechlorination, *Desulfitobacterium* sp. strain PR (Ding et al., 2014) and *Dehalobacter* sp. strain UNSWDHB (Deshpande et al., 2013), a *cfrA*/*dcrA*-like *rdhA* gene was identified, whose corresponding protein shares >94% amino acid identity to CfrA or DcrA. In *Desulfitobacterium* sp. strain PR, the *rdhA* gene was named *ctrA* (Accession #AGO27983); however, it encodes an RDase that, unlike CfrA and DcrA, dechlorinates all three substrates, CF, 1,1,1-TCA and 1,1-DCA (Ding et al., 2014). Because of the natural occurrence of chloroform and the discovery of other chloroform-respiring organisms, it seems more likely that the last common ancestor to strains CF and DCA was a chloroform-respiring organism. The structural similarity between CF and 1,1,1-TCA probably enabled this organism to use 1,1,1-TCA present in the groundwater where the ACT-3 enrichment culture originated (Grostern and Edwards, 2006a). The differentiation of strains CF and DCA may have been initiated by random sequence variations between the *cfrA* and *dcrA* genes resulting in modified substrate preference in the corresponding enzymes that eventually led to the complete specialization for 1,1,1-TCA or 1,1-DCA. The underlying reason for the selective advantage of having these two functions in separate organisms rather than in one is not known. Similar case of such specialization within an enrichment culture was reported in a tetrachloroethene-dechlorinating bacterial consortia (Buttet et al., 2013), where each of two *Sulfurospirillum* populations harbor a very similar but distinct PceA enzyme with different substrate specificity to chloroethenes. Strain differentiation was also observed in *Dehalobacter* populations dechlorinating different dichlorobenzene isomers (Nelson et al., 2014).

### Two *rdhA* clusters in *Dehalobacter* genomes

Similar to *Dehalococcoides* genomes, *Dehalobacter* genomes possess multiple, non-identical *rdhA* genes (Table 1). In *Dehalococcoides* genomes, most of the *rdhA* genes are located in two high plasticity (HP) regions associated with hot recombination sites including some tRNA genes and the tmRNA gene *ssrA* (McMurdie et al., 2009). Similarly, in *Dehalobacter* genomes, many *rdhA* genes (21/25 in strain PER-K23, 10/17 in strain CF and 10/17 in strain DCA) cluster into two small regions designated cluster 1 and cluster 2 (Figure 1) that are conserved in these three genomes. However, unlike the *Dehalococcoides* HP regions, neither of these *Dehalobacter* regions includes direct repeats indicating recent insertion events, or DNA recombinases, except for some transposases. Moreover, there is no evidence that these transposases form composite transposons with *rdhA* genes, as seen in a transposon in *Desulfitobacterium* (Maillard et al., 2005; Duret et al., 2012). No tRNA genes or other hot recombination sites are located near these two *rdhA* clusters. The formation of these two *rdhA* clusters in *Dehalobacter* genomes thus seems unrelated to site-specific sequence recombination events or genomic islands as seen in *Dehalococcoides* genomes.

Sequence duplication may have played a role in the development of these two *rdhA* clusters. We constructed a phylogenetic tree of all *rdhA* genes found in the genomes of strains CF, DCA, PER-K23, UNSWDHB, and E1 (Figure S3). Of the 25 *rdhA* genes in strain PER-K23 many are similar to each other (Figure S3). For example, two *rdhA* genes (Dehre_2031 and Dehre_2044) share an amino acid sequence identity of 98.2% and a nucleotide identity of 97.5%. Moreover, similar *rdhA* genes are often close to each other in the genome within the two *rdhA* clusters (Figure S4). A similar scenario exists in the complete genomes of strains CF and DCA. Analysis of the two *rdhA* clusters in these genomes revealed the presence of highly similar regions (nucleotide similarity ranging from 70 to 92%; pair-wise blocks in Figure S4), potentially resulting from sequence duplication events.

### Insertion sequence transposition

Transposition events (TEs) are known to have positive, neutral and negative effects on the host (Rebollo et al., 2012). TEs may be generally beneficial to the whole population despite deleterious effects on individuals (Rebollo et al., 2012). The comparison of the complete genomes of strain CF, strain DCA, and strain PER-K23 reveals many strain-specific transposition events and some of them clearly show gene disruptions (data not shown). However, most TEs detected in these three *Dehalobacter* genomes reside in non-coding regions and therefore their effects cannot be evaluated without more knowledge of gene regulation. Since TEs typically exist as interspersed repeats (direct or inverted) in a genome, they are hot genome rearrangement sites via intra-genomic homologous recombination.

Several lines of evidence exist to suggest that insertion sequence (IS) transposition has played an important role in shaping *Dehalobacter* genomes. A large number of ISs were found in the three complete *Dehalobacter* genomes (68, 71, and 69 in strains CF, DCA, and PER-K23, respectively), and many strain-specific transposition events were identified when aligning the genomes of strains CF and DCA. In the genome of strain CF, 27 distinct ISs were identified, 14 of which exist in more than one copy in the CF genome; an extreme case is that of IS (DCF50_p264) that exists in nine copies in the genome. While strains CF and DCA share all ISs, their copy numbers vary in some instances, revealing many strain-specific transposition events that have happened since the differentiation of the two strains (Figure 2) The genome of strain PER-K23 has 25 distinct ISs, of which five are shared with strain CF.

### Homologous recombination

**Figure 4:**
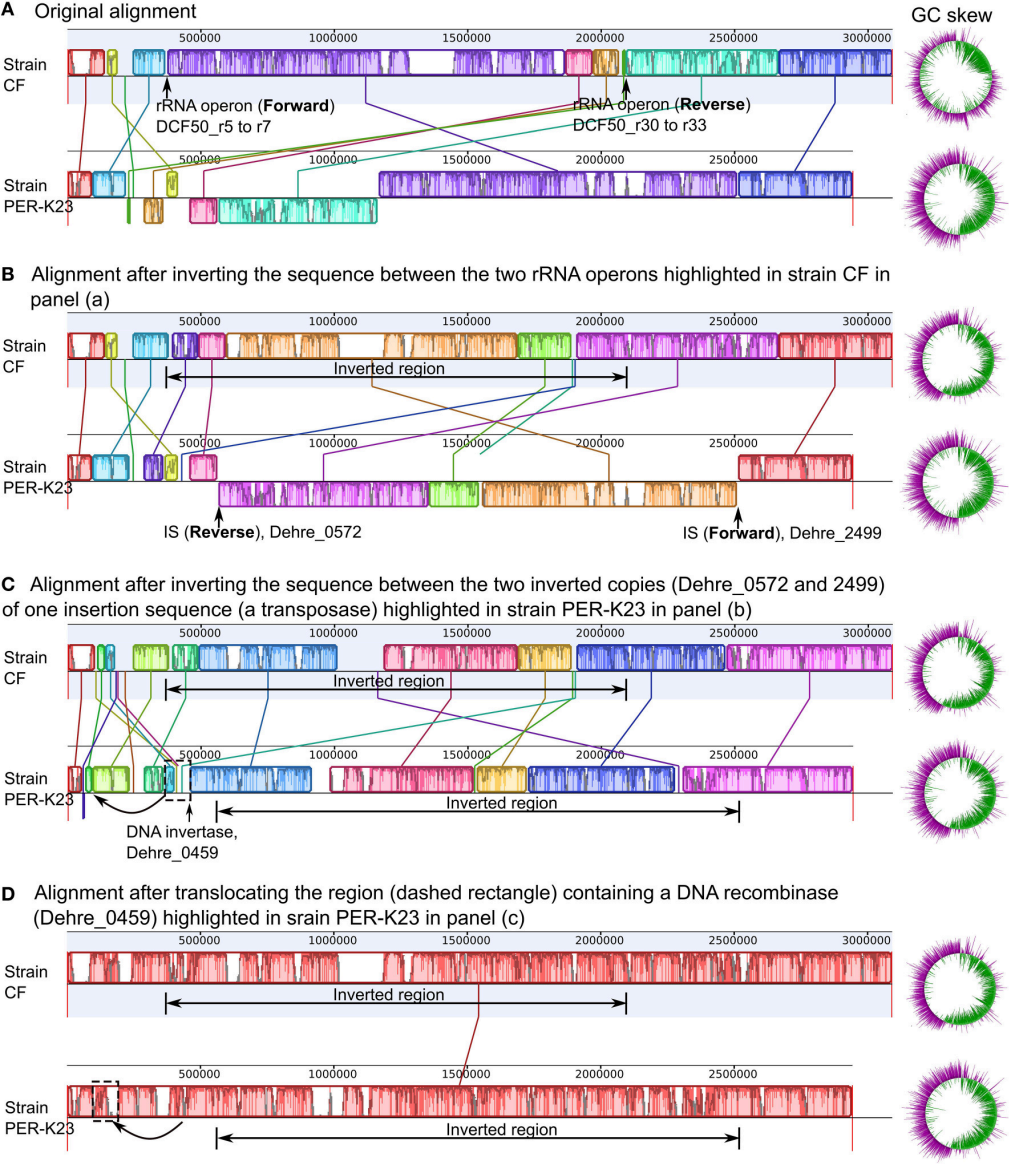
**Three potential genome rearrangements between *Dehalobacter restrictus* strain PER-K23 and *Dehalobacter* sp. strain CF**. Note the blocks with identical color represent corresponding areas between the genomes of strain CF and strain PER-K23 with high similarity. Panel **(A)** shows the Mauve alignment and original GC skew profile of the two genomes. Panels **(B–D)** represent the 3-step artificial reversion scenario that could explain the genome rearrangements observed between the two strains. Steps 1 and 2 in Panels **(B, C)** describe two sequence inversions, and step 3 in Panel **(D)** represents a translocation. The resulting changes in Mauve alignments are depicted with corresponding changes in GC skew profiles shown to the right.

Genes essential for homologous recombination were found in all *Dehalobacter* genomes including *recA, recFOR* and *ruvABC* (Table S1; tab 41). The presence of a large number of intra-genomic repeats including rRNA operons and ISs provides potential recombination sites for intra-genomic homologous recombination, contributing to genome plasticity. Three copies of the rRNA operons exist in the genomes of strains CF and DCA, while strain PER-K23 genome has four. As mentioned earlier, the strain CF (or DCA) genome has a global GC-skew profile markedly different from that of strain PER-K23 (Figure 1). Three intra-genomic sequence rearrangement events could explain the difference (Figure 4). Two of these rearrangements are homologous recombination of inverted repeats (Treangen et al., 2009): (i) a sequence inversion between two inverted rRNA gene operons (shown in Figure 4A, with end result Figure 4B) and (ii) a sequence inversion between two inverted copies of an IS, Dehre_0572 and Dehre_2499 (shown in Figure 4B with end result in Figure 4C). Finally, a third translocation event, likely related to a DNA recombinase, Dehre_0459 (shown in Figure 4C), would result in complete synteny between these two genomes (Figure 4D). Sequence inversion events catalyzed by inverted rRNA operons have been reported in *Escherichia coli* (Hill and Gray, 1988). This sequence inversion could have happened in either *Dehalobacter* genome as they both share three rRNA operons. However, this event is more likely to have happened in the genome of strain CF because the reversion of this event results in a genome with a more symmetrical GC-skew (Figure 4). Sequence evidence supporting the two other genome rearrangements based on ISs (Dehre_0572 and Dehre_2499) and the DNA recombinase (Dehre_0459) was found only in the genome of strain PER-K23. Therefore, these two events were more likely to have occurred in strain PER-K23.

Beyond the two *rdhA* clusters, major sequence variations between *Dehalobacter* strains were observed in three other regions of these genomes, labeled regions A, B, and C in Figure 1 and A1/A2, B1/B2, C1/C2 in Figure 2. Here we highlighted some interesting observations around region B1/B2. Although region B1 and B2 mostly consist of non-homologous sequences, their upstream and downstream neighborhoods are orthologous, but differ by intensively distributed SNPs. It is unreasonable to assume that the intensive SNPs in these regions are caused by the accumulation of single nucleotide mutations since most other orthologous regions between these two genomes are completely identical. When comparing closely related *Dehalobacter* genomes (CF vs. DCA, CF vs. UNSWDHB, and E1 vs. PER-K23; Figure S5), we observed the same phenomenon, i.e., that some large, continuous orthologous regions between pairs of genomes have nearly no SNPs, while others contain a large numbers of SNPs, with a large proportion of synonymous mutations (Figure S5). If these SNPs accumulated through single nucleotide mutations, they should be more evenly and randomly distributed along the whole genomes. Thus *Dehalobacter* genomes seem to accumulate changes by larger insertions or deletions more extensively than by SNPs, which suggest that mechanisms such as homologous recombination are more significant than point mutations in shaping *Dehalobacter* genomes. Interestingly, similar phenomena were found between *Desulfitobacterium* genomes, but were not found when we compared *Dehalococcoides* genomes, in which sequence variations are more evenly distributed across the genome (data not shown).

### Horizontal gene transfer

Assemblies resembling the Type IV pilus have been implicated in foreign DNA uptake in some bacteria (Chen and Dubnau, 2004). In *Dehalobacter* genomes, many genes involved in type IV pilus assembly were identified (Table S1; tab 40). Additional evidence for horizontal gene transfer in *Dehalobacter* stems from the similarity between *pceABCT* operons identified in *Dehalobacter and Desulfitobacterium* strains (Maillard et al., 2005; Duret et al., 2012). In *Desulfitobacterium*, the *pceABCT* operon is located in the Tn-Dha1 transposon flanked by two direct copies of an IS. The high similarity of this operon with the *pceABCT* operon identified in *Dehalobacter restrictus* strain PER-K23 suggested horizontal gene transfer (Maillard et al., 2005; Duret et al., 2012). This *pceABCT* operon is conserved in the same genome context in all five *Dehalobacter* genomes, although considerable sequence variations in this operon exists between these five *Dehalobacter* strains, indicating that the *pceABCT* operon has been carried by each *Dehalobacter* for some time (Figure S6). Interestingly, in the genomes of strain CF and strain DCA, this operon is located in a region flanked by two direct repeats (~400 bp each) with other repeat patterns, indicating that the operon could have been acquired horizontally. These repeat patterns were not found in strain PER-K23 nor in strain E1. No other evidence for the presence of genomic islands or horizontal gene transfer events involving *rdhA* genes was found in any of the five *Dehalobacter* genomes.

Horizontal gene transfer appears to play an important role in shaping *Dehalobacter* genomes. As indicated previously, major sequence variations between the five genomes are located in regions A, B, and C highlighted in Figure 1. Region A features a recent insertion of a 64 kb fragment (A1) in the genome of strain CF (Figure 2). This region has a relatively low G+C content (40%) and most genes involved are unique to strain CF. The insertion is likely related to a phage integrase (DCF50_p74) located at the 3′ end of A1 (Figure 2). In Region C, which has a low G+C content (35%), strain CF has many phage-related genes. Incorporation of region C1 (Figure 2) into strain CF is likely related to a site-specific recombinase (DCF50_p1382) located at the 5′ end of C1. On the other hand, sequence C2 from strain DCA is ~79 kb long and has a G+C content of 45.2%, similar to the average G+C content of the whole genome, and may indicate that C2 is native to *Dehalobacter*. In region B, both B1 (strain CF) and B2 (Strain DCA) have a low G+C content, and poor sequence conservation was found between all five *Dehalobacter* genomes, indicating a hypervariable region. Region D (Figure 1) is another large phage–related region shared by both strain CF and DCA, but not found in strain PER-K23 and E1. Region D is ~43 kb long and contains many genes encoding either phage-related or hypothetical proteins, including a phage integrate located at the 3′ end of the region. This insertion event targeted tRNA-Thr (DCF50_r14) and resulted in duplication (45 bp) at the insertion site, which contains a partial tRNA-Thr gene.

### Metabolic potential

We examined in detail the gene annotations of *Dehalobacter* to reveal metabolic potential and any differences between the strains. Overall, these *Dehalobacter* strains are highly similar. Genes for all major metabolic pathways and physiological requirements of *Dehalobacter* strain CF grouped by pathway or category are compiled and organized in Excel Table S1 (42 categories/tabs in total), together with corresponding orthologs identified from *Dehalobacter* strains DCA, UNSWDHB, PER-K23, and E1. Corresponding orthologs from *D. hafniense* strain Y51 and *D. mccartyi* strain 195 are also included in Table S1 for reference to a close relative (*Desulfitobacterium*) as well as the obligate organohalide-respiring genus *Dehalococcoides*. Relevant gene expression information is also included based on a recent proteomic study of *Dehalobacter restrictus* strain PER-K23 (Rupakula et al., 2013). A Table of Contents for all the categories of cataloged genes is provided in the first sheet of the Table S1 Excel file. The following discussion of metabolic pathways applies to all five *Dehalobacter* strains unless specifically noted. Only locus tags for strain CF are referenced in the text; corresponding orthologs in other genomes are readily available in Table S1.

### Energy metabolism

Strain PER-K23 is the best characterized *Dehalobacter* isolate and was shown to be an obligate dechlorinator. However, all five *Dehalobacter* genomes have a complete glycolysis pathway, and genes involved in various catabolic pathways such as genes encoding alcohol dehydrogenase (DCF50_p2281), aldehyde dehydrogenase (DCF50_p2406), 6-phosphofructokinase (DCF50_p872), pyruvate kinase (DCF50_p653), pyruvate formate lyase (DCF50_p2283 and p2284), and formate hydrogen lyase (DCF50_p760 to p766). This suggests that *Dehalobacter* have the potential to ferment or use electron donors other than H_2_ or formate. All of these genes have orthologs in *D. hafniense* strain Y51, and most *Desulfitobacterium* strains are capable of pyruvate fermentation. Other than *rdhA* genes, no genes for other electron–accepting reactions were identified in any of the *Dehalobacter* genomes. Only putative cytochrome b genes were found. Thus, considering the presence of 86 *rdhA* genes in these five genomes, and lack of fermentative growth, these *Dehalobacter* strains appear specialized for organohalide respiration (Holliger et al., 1998).

### Central carbon metabolism

The characterization of the three previously isolated *Dehalobacter* strains (Wild et al., 1996; Holliger et al., 1998; Sun et al., 2002) showed that acetate is essential for growth, but cannot support organohalide respiration without hydrogen or formate. The incorporation of acetate likely starts with acetate–CoA ligase (DCF50_ p435) to make acetyl-CoA, which then combines with carbon dioxide (CO_2_) to produce pyruvate using pyruvate-flavodoxin oxidoreductase (Dcf50_ p269 or p2740). Heterotrophic CO_2_ fixation is supported by some ^14^CO_2_ fixation experiments with strain PER-K23 (Holliger et al., 1993). Genes for a complete Wood–Ljungdahl pathway were found in the genomes of strains CF, DCA, and PER-K23 (Figure S7A), and all of these genes, except 5,10-methylenetetrahydrofolate reductase (Dcf50_p297 or Dehre_0155), were found expressed in strain PER-K23 (Rupakula et al., 2013). Genes required for a functional Wood-Ljungdahl pathway were also found in *Desulfitobacterium* genomes and it is known that *D. hafniense* strain DCB-2 can grow on H_2_ and CO_2_ via reductive acetogenesis (Kim et al., 2012). However, no known *Dehalobacter* isolates have been able to grow only with H_2_ and CO_2_ (Wild et al., 1996; Holliger et al., 1998; Sun et al., 2002), indicating that the Wood-Ljungdahl pathway plays a different role in *Dehalobacter*. The role of this pathway is even more questionable since it is incomplete in strain E1 as a result of sequence deletion (Figure S7A).

**Figure 5:**
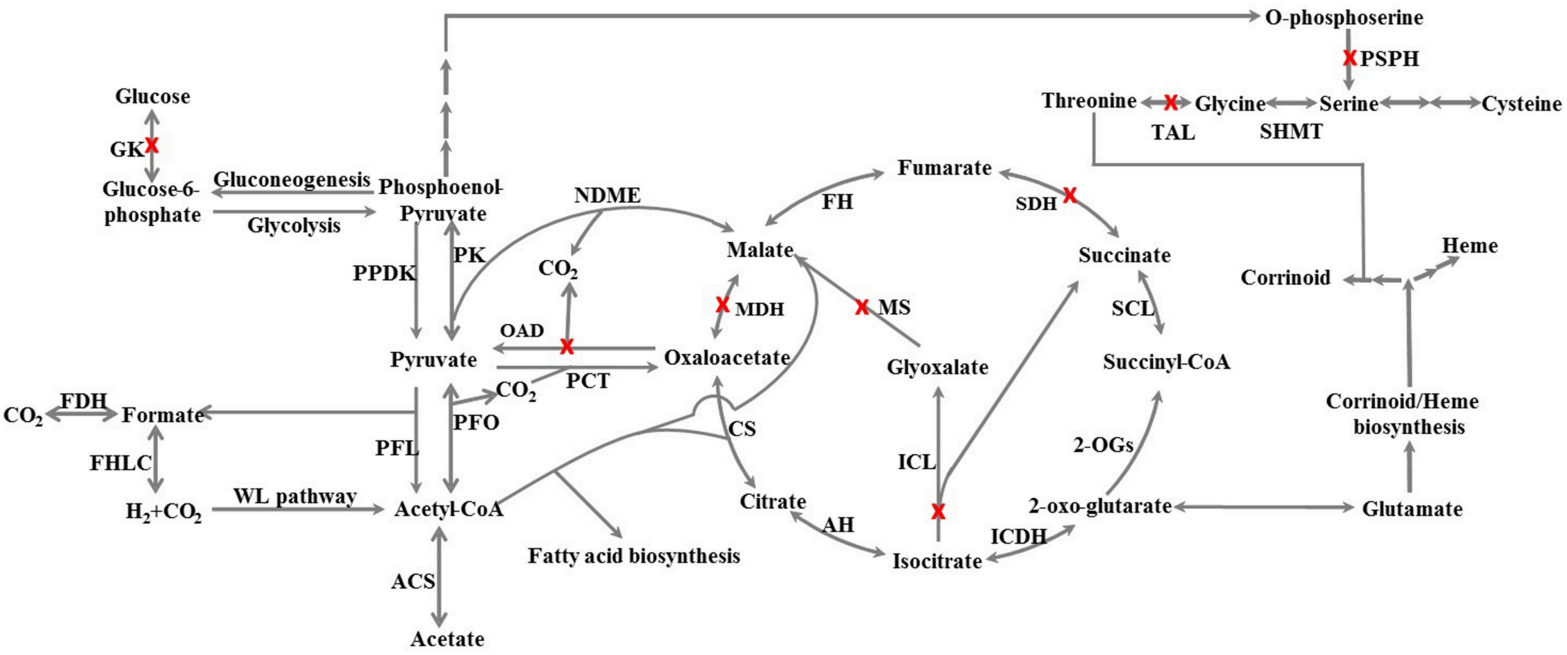
**Schematic of the central carbon metabolism and corrinoid/heme biosynthesis of *Dehalobacter* sp. strain CF inferred from genome annotation**. Enzymes involved in the reactions are listed below with full name and gene locus_tags from strain CF: PK, pyruvate kinase, p653; PPDK, pyruvate, phosphate dikinase, p2541; FDH, formate dehydrogenase, p924-927; FHL, formate hydrogenlyase, p760-766; ACS, acetate:CoA ligase (AMP-forming), p435; PFL, pyruvate formate-lyase, p2283 + p2284; PFO, pyruvate-flavodoxin oxidoreductase, p269 or p2740; PCT, pyruvate carboxyl transferase, p2314; NDME, NADP-dependent malic enzyme, p397; CS, citrate synthase (si), p2711 or p2808; AH, aconitate hydratase, p995; ICDH, isocitrate dehydrogenase [NADP], p1096; 2-OGs, 2-oxoglutarate:ferredoxin oxidoreductase, p978-981; SCL, succinate:CoA ligase, p393 + p395; FH, fumarate hydratase, p2651 + p2652; SMHT, serine hydroxymethyltransferase, p2888. A red X on a pathway means the gene for the enzyme is not found. GK, glucokinase; OAD, oxaloacetate decarboxylase; PSPH, phosphoserine phosphatase; SDH, succinate dehydrogenase; MDH, malate dehydrogenase; ICL, isocitrate lyase; MS, malate synthase; TAL, threonine aldolase.

Many anaerobes have an incomplete tricarboxylic acid cycle (TCA cycle), which mainly serves anabolic purposes. For example, *Dehalococcoides* strains have an incomplete TCA cycle (Ahsanul Islam et al., 2010; Marco-Urrea et al., 2011). The TCA cycle of strains CF and DCA appears incomplete as well, missing genes for malate dehydrogenase, succinate dehydrogenase, and for the glyoxylate bypass (Figure 5). However, the loss of malate dehydrogenase can be compensated for by the presence of an NADP–dependent malic enzyme (DCF50_p397). Despite being incomplete, all intermediates within the cycle can be synthesized from pyruvate and acetyl-CoA with an oxidative half-cycle to succinate and a reductive half-cycle to fumarate (Figure 5). Moreover, all of these enzymes were found expressed in strain PER-K23 (Table S1; tab 2). *D. hafniense* strain Y51 and DCB-2 also appear to have an incomplete TCA cycle, but is missing a different step, 2-oxoglutarate dehydrogenase (Nonaka et al., 2006; Kim et al., 2012), which is present in these five *Dehalobacter* genomes (DCF50_p978 to p981).

### Amino acid metabolism

**Figure 6:**
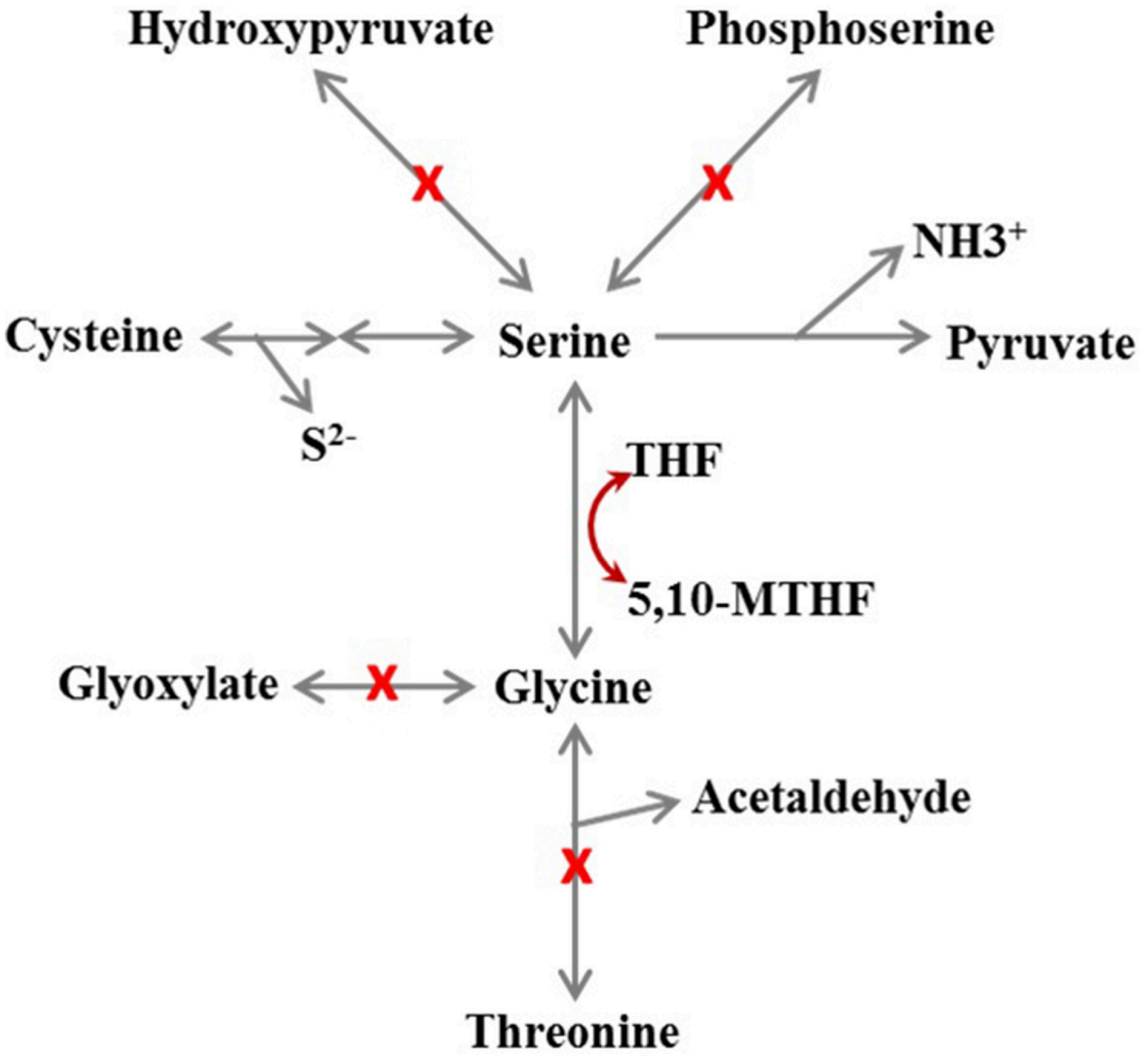
**Schematic of the metabolic defects in amino acid biosynthesis of *Dehalobacter* sp. strain CF inferred from genome annotation**. THF, tetrahydrofolate; 5,10-MTHF, 5,10- methylene-tetrahydrofolate. A red X on a pathway means the gene encoding the enzyme was not found. The Figure shows that either glycine, cysteine, or serine is needed because all other routes for serine biosynthesis are missing.

The genome annotation suggests that all *Dehalobacter* genomes possess complete biosynthesis pathways for all amino acids except serine, and the enzymes involved in these pathways were detected by proteomic analysis in strain PER-K23 (Table S1; tabs 7-16). From the genome annotation, *Dehalobacter* strains seem not be able to synthesize serine since the gene encoding phosphoserine phosphatase (EC 3.1.3.3), the enzyme that catalyzes the last step in serine biosynthesis, is missing from all five *Dehalobacter* genomes (Figure 6). Although *Dehalobacter* can acquire serine from glycine by serine hydroxymethyltransferase (EC 2.1.2.1), the gene encoding L-threonine aldolase (EC 4.1.2.5), which converts L-threonine to glycine, is also not present in the genomes of strains CF and DCA (Figure 6). This defect in serine biosynthesis can also hamper the biosynthesis of cysteine and glycine (Figure 6). It is unclear if *Dehalobacter* strains are able to salvage serine from pyruvate through the reverse reaction of L-serine dehydratase (EC 4.3.1.17). An alternative salvage mechanism must operate in *Dehalobacter* cells since the growth of strain PER-K23 only requires arginine, histidine, and threonine but not cysteine, glycine, or serine (Holliger et al., 1998).

### Organic cofactors and their precursors

The biosynthesis pathways for essential cofactors, including terpenoid, menaquinone, riboflavin, nicotinamide adenine dinucleotide, folate, pantothenate, and pyridoxal phosphate, appear complete in all *Dehalobacter* genomes as well, while the typical biosynthesis pathways for two essential cofactors, thiamin and biotin, appear absent (Table S1). The presence of complete biosynthesis pathways for menaquinone and its terpenoid backbone, octaprenyl diphosphate, agrees with a previous finding that menaquinones are the only quinones detected in the cell biomass of *Dehalobacter* strain PER-K23 (Holliger et al., 1998). Menaquinone has been proposed to serve as an electron mediator between hydrogenases and RDases in strain PER-K23 (Schumacher and Holliger, 1996). Biotin has an essential role in lipid biosynthesis. The absence of a biotin biosynthesis pathway among these *Dehalobacter* genomes is surprising since strain PER-K23 does not require biotin for growth (Holliger et al., 1998). The absence of a thiamin biosynthesis pathway among all the *Dehalobacter* genomes is consistent with the fact that the growth of strain PER-K23 requires a thiamin supplement (Holliger et al., 1998).

Corrinoids are essential cofactor of RDases. The completeness of the corrinoid biosynthesis pathway varies among these *Dehalobacter* strains. While the pathway is complete in strains CF, DCA and UNSWDHB, an essential gene appears to be truncated in strain PER-K23 (Rupakula et al., 2015) and several genes are absent in strain E1 due to sequence deletion events (Figure S7C). Most genes involved in this pathway reside in two gene clusters: genes involved in the upper pathway (from glutamyl-tRNA to cobyrinate) are located in one cluster (DCF50_p2930 to p2943 in strain CF) and genes in the lower pathway are located in another cluster (DCF50_p799 to p808 in strain CF; Table S1). In strain PER-K23, a 101-bp sequence deletion within the coding region of cobalt-precorrin-3b C17-methyltransferase (CbiH, Dehre_2856) results in a frame shift mutation; this gene was annotated as a pseudogene (Rupakula et al., 2013). In addition to CbiH, several enzymes from the same upper pathway gene cluster were not detected in the proteome of strain PER-K23 (Rupakula et al., 2013). A recent study suggested that one operon encoding essential genes of corrinoid biosynthesis is upregulated both at the transcriptional (346-fold) and proteomic level (46-fold on average) upon cobalamin starvation in strain PER-K23 (Rupakula et al., 2015), which suggests its biosynthesis pathway is at least partially functional. The disruption of *cbiH* may explain why the growth of strain PER-K23 requires cobalamin supplement (Holliger et al., 1998). In strain E1, there is a larger sequence (~6 kb) deleted at the 3′ end of the same gene cluster (the upper pathway), resulting in the loss of genes encoding cobalt-precorrin-4 C11-methyltransferase (CbiF), precorrin-2 C20-methyltransferase (CbiL), cobalt-precorrin-6 synthase (CbiD), and four subunits of a cobalt ECF transporter (CbiOQNM; Figure S7C). These genes were not found elsewhere in the draft genome of strain E1. Strain E1 exist in a co-culture with a *Sedimentibacter* strain, which has a complete corrinoid biosynthesis pathway, thus the inability to produce corrinoid has been proposed as an explanation for the dependence of strain E1 on this partner population (Maphosa et al., 2012).

Heme (heme b) is a cofactor of cytochrome b, a subunit of hup-type Ni,Fe-hydrogenases found in all five *Dehalobacter* genomes (Table S1). However, two genes involved in the classic heme biosynthesis pathway (Layer et al., 2010), which starts from uroporphyrinogen III to heme via coproporphyrinogen III and other intermediates were not found in any of the five *Dehalobacter* genomes. It seems that *Dehalobacter* likely use the recently described anaerobic heme biosynthesis pathway found in archaea and sulfate-reducing bacteria (Kuhner et al., 2014). In this pathway, heme is synthesized from uroporphyrinogen III via the intermediates precorrin-2, sirohydrochlorin, siroheme, 12,18-didecarboxysiroheme, and iron-coproporphyrin III (Kuhner et al., 2014). In *Dehalobacter*, siroheme is likely synthesized from uroporphyrinogen III by CysG (DCF50_p2935), a multifunction enzyme (EC1.3.1.76, 2.1.1.107, and 4.99.1.4). Then, the newly described alternative pathway likely catalyzes the steps from siroheme via iron-coproporphyrin III to heme, as was recently demonstrated in the methanogenic archaeon *Methanosarcina barkeri* (Kuhner et al., 2014). Unlike the classic protoporphyrin–dependent pathway, this alternative pathway “hijacks” siroheme from the heme d1 biosynthetic pathway to synthesize heme via six successive decarboxylation reactions by decarboxylase AhbAB (or NirDL/H) (Mbar1459 and 1460), AhbC (or NirJ2) (Mbar1473), and AhbD (or NirJ1) (Mbar1458; Bali et al., 2011; Kuhner et al., 2014). Three proteins were found in all five *Dehalobacter* genomes showing high amino acid identity with these four proteins: DCF50_1080: 44% a.a. identity to a fusion of AhbA and AhbB, DCF50_1081: 40% a.a. identity to AhbD, and DCF50_1082: 55% a.a. identity to AhbC, suggesting this pathway is also operative in *Dehalobacter*.

Molybdopterin is a cofactor incorporating molybdenum and tungsten and has essential roles for specific enzyme functions (Kisker et al., 1997). Two selenocysteine-containing and molybdopterin-binding formate dehydrogenases (Dcf50_p924 to p927 and Dcf50_p1622 to p1626) are encoded in the five *Dehalobacter* genomes. These two formate dehydrogenases were found expressed in strain PER-K23 (Rupakula et al., 2013) even though it cannot use formate as an electron donor for organohalide respiration. In contrast, *Dehalobacter* strain TCA1 was reported to use both H_2_ and formate as electron donors (Sun et al., 2002). Most genes involved in the molybdopterin biosynthesis pathway are located in one gene cluster (Dcf50_p1639 to p1645) (Figure S7B). However, the genes encoding molybdopterin synthase (MoaD and MoaE) were not found. Instead, in strain PER-K23 and strain E1, two MOSC domain-containing proteins (Dehre_2363 and Dehre_2365, 30% a.a. identity) were found in the same gene cluster. MOSC is a predicted sulfur-carrier domain that delivers sulfur for the formation of diverse sulfur-metal clusters (Anantharaman and Aravind, 2002). It is possible that these two proteins fulfill the function of molybdopterin synthase. The ortholog of one MOSC domain-containing protein (Dehre_2365) was not found in either strain CF or strain DCA probably due to sequence deletion (Figure S7B). These observations do not explain why some strains cannot use formate as an electron donor.

### Anabolic pathways

Many complete anabolic pathways were identified in all genomes, including gluconeogenesis, pentose pathways, and biosynthesis pathways for fatty acids, purines, pyrimidines, amino sugars, and peptidoglycan (Table S1). It is not surprising that a large number of genes that distinguish *Dehalobacter* and *Desulfitobacterium* from *Dehalococcoides* belong to peptidoglycan biosynthesis, sporulation, and flagella biosynthesis (chemotaxis). Strain PER-K23 has a peptidoglycan-containing cell wall (Holliger et al., 1998) and all known *Dehalobacter* isolates have at least one flagellum (Wild et al., 1996; Sun et al., 2002). *Dehalobacter* have never been shown to form spores. Strains CF and DCA have two gene clusters with metabolic functions that were not found in strains PER-K23 and E1: one (DCF50_p2009 to p2020) encodes a complete nitrogen fixation operon (Table S1, tab34) including nitrogenase complex genes nifKDH (DCF50_p2016-p2018) and the other (DCF50_p194 to p200) encodes some genes (Table S1, tab39) related to arsenate resistance or detoxification. The differences between the genomes in these two gene clusters appear to be caused by sequence insertion or deletion.

### Motility, chemotaxis, and regulation of organohalide respiration

*Dehalobacter* genomes possess the full complements of genes encoding the flagellar apparatus and the chemotaxis cascade (Table S1, tabs 32-33; Wadhams and Armitage, 2004). This is consistent with the observation of flagella within *Dehalobacter restrictus* strain PER-K23 (Holliger et al., 1998). Chemotactic behavior is mediated by both one and two-component signal transduction systems that coordinate downstream effectors of gene regulation and motility. The presence of signal transducers and transcriptional regulators near *rdhA* genes (Table S1, tab 42) in these five *Dehalobacter* genomes suggests that reductive dechlorination is regulated. The search for protein domains associated with signal transduction and gene regulation within five protein-coding genes upstream or downstream of putative *rdhA* genes in representative genomes of *Dehalobacter, Desulfitobacterium*, and *Dehalococcoides* revealed the presence of group-specific transcriptional regulators (Table S2).

The genomes of the *Firmicutes Dehalobacter* and *Desulfitobacterium* contain abundant cNMP_binding and HTH_Crp_2 domain-containing proteins near *rdhA* genes (Table S2). Specifically, the cNMP_binding domain proteins contain a 120 amino acid-long protein motif diagnostic for members of the Crp/Fnr family (Weber et al., 1987; Körner et al., 2003). Crp/Fnr transcription factors are a diverse group of regulators that enact a response by repressing or enhancing gene transcription by binding to a gene's promoter in the presence of a small metabolite (Smidt et al., 2000; Santos et al., 2009). The genes *cfrA* and *dcrA*, encoding the functionally expressed RDases in strains CF and DCA each possess a nearby gene encoding a Crp/Fnr transcriptional regulator. However, the Crp/Fnr transcriptional regulator near *dcrA* has been truncated by a putative transposase-encoding gene. Still, there are 16 additional *rdhA* gene neighborhoods in strain DCA that encode highly similar Crp/Fnr transcriptional regulators that may compensate for the truncated gene. A Crp/Fnr ortholog, CprK, in *Desulfitobacterium dehalogenans* has been shown to influence transcription of a gene involved in respiration of ortho-substituted chlorophenolic compounds (Pop et al., 2004). The crystal structure of CprK dimer has been solved and the molecular basis for transcriptional regulation has also been proposed (Levy et al., 2008; Kemp et al., 2013). These experiments showed that the transcriptional activation is associated with the salt bridge interaction between the chlorinated substrate and the strictly conserved K133 residue. The binding of the chlorinated substrate to the N-terminal sensor domain can induce an allosteric effect on the C-terminal DNA-binding domain. Furthermore, CprK displays remarkable ability to distinguish between chlorinated substrates and dechlorinated products with a 10^4^ difference in distinguish between chlorinated substrates and dechlorinated products with a 10^4^ difference in affinity.

In contrast, many regulatory proteins near *rdhA* genes in the genomes of the *Chloroflexi* genera *Dehalococcoides* and *Dehalogenimonas* contain response regulator (Response_reg, Trans_reg_C) and associated histidine kinase (HisKA, ATPase_c) or sensor (PAS) domains (Table S2). The presence of genes encoding proteins with histidine kinase-response regulator (HK-RR) domains near *rdhA* genes in the *Dehalococcoides* and *Dehalogenimonas* genomes suggests a role for the associated proteins in controlling organohalide respiration in this phylogenetic group, as previously proposed (Seshadri et al., 2005). Some transcriptional regulators adjacent to *rdhA* genes in organohalide-respiring *Chloroflexi* encode proteins containing MarR_2 domains (Table S2). MarR regulators are known to be involved in non-specific antibiotic resistance in *Salmonella typhimurium* and *E. coli*, and are expressed in the presence of these compounds (Sulavik et al., 1995, 1997). MarR transcription factors have also been implicated in the regulation of genes in response to exposure to phenolic compounds (Sulavik et al., 1995). A recent study indicated that MarR may act as a repressor of *rdhA* gene transcription in *Dehalococcoides*, and is activated in the presence of specific dibenzo-p-dioxins (Wagner et al., 2013).

The clear distinction in the types of transcriptional regulators present in genomic regions surrounding *rdhA* genes in organohalide-respiring *Firmicutes* and *Chloroflexi* indicates that the two phylogenetically distinct groups have converged on the same physiology via distinct evolutionary paths. The genome analysis herein confirms the distinct inherited traits of these two very different phylogenetic groups, despite very similar niche-specific metabolism and function.

## Materials and methods

### Genome assembly and annotation

The assembly of the two *Dehalobacter* genomes of strains CF and DCA was reported in a previous publication (Tang et al., 2012). The gene annotation of strain CF was performed with two automatic genome annotation pipelines: RAST (Aziz et al., 2008) and IMG-ER (Markowitz et al., 2009), separately. The subsequent results from the two annotation pipelines were compared and combined with inconsistencies resolved by manual curation. Some annotations were manually refined based on the analyses of sequence homology and genome context. The genes of strain DCA were first annotated with RAST, and those sharing high identity (50% amino acid identity) with genes in strain CF were examined and curated to keep consistency, if needed. The annotation of strain PER-K23 was retrieved from IMG (http://img.jgi.doe.gov/) with Taxon Object ID of 2510065016 (Rupakula et al., 2013). The draft genome of strain E1, consisting of 102 contigs, was retrieved from GenBank with the accession number of CANE00000000. Genes in the draft genome were identified with Glimmer 3 (Delcher et al., 2007), accessed through Geneious pro v. 6.1.4 (Drummond et al., 2011). The annotation of some genes of interest in strain E1 was performed by manual BLASTP against NCBI databases. Whole genome alignment between strains CF, DCA, and PER-K23 was performed by the Mauve alignment (Darling et al., 2010) in Geneious pro. DNA sequence alignments of large genome regions (containing multiple genes) were extracted from the results of Mauve alignment using the option “Extract Mauve Regions” in Geneious pro; Default settings were used in most cases. Multiple sequence alignments of short DNA sequences, such as single genes, were performed by MUSCLE (Edgar, 2004) within Geneious pro. Genome circular maps were created with BRIG (Alikhan et al., 2011). DNA sequence repeats were identified with Repseek (Achaz et al., 2007) and inverted repeats were identified with Inverted Repeat Finder (Warburton et al., 2004). Orthologs between *Dehalobacter* genomes were identified with reciprocal BLASTP with an *e*-value of 1E-10. Orthologs between *Dehalobacter* sp. strain CF, *D. hafniense* strain Y51, and *D. mccartyi* strain 195 were identified with reciprocal BLASTP with *e*-value of 1E-5.

Genome protein annotations used in the transcriptional regulator analysis were collected from either RAST (rast.nmpdr.org) or IMG (img.jgi.doe.gov) for use with the pfam_scan.pl script (Finn et al., 2010). The script was run with default settings and Pfam domains were identified from the Pfam-A database only. Using custom Perl scripts, the output was parsed into associated transcriptional regulatory domains and general functional categories based on the MIST2 database (www.mistdb.com) signaling domains (Ulrich and Zhulin, 2009). A combination of genome viewers (RAST, IMG) and blast analysis of genome FASTA files from NCBI were used to discover *rdhA* genes and their surrounding putative regulators. Each coding region less than 5 protein-coding genes upstream or downstream of the catalytic reductive dehalogenase subunit was included as part of the reductive dehalogenase gene neighborhood. Hypothetical or putative genes were counted only if they contained significant scores (less than default 0.001 domain e-value) for Pfam protein domains associated with signal transduction and gene regulatory activity as defined by the MIST2 database (Ulrich and Zhulin, 2009).

## Author contributions

ST assemblies the Dehalobacter genomes. ST and PW annotate the Dehalobacter genomes. EE, ST, PW, and SH analyze the data. ST, PW, FL, and EE write the paper.

### Conflict of interest statement

The authors declare that the research was conducted in the absence of any commercial or financial relationships that could be construed as a potential conflict of interest.
