# Supplementary material for: Sister Dehalobacter Genomes Reveal Specialization in Organohalide Respiration and Recent Strain Differentiation Likely Driven by Chlorinated Substrates
Source: Front Microbiol. 2016 Feb 12;7:100. doi: 10.3389/fmicb.2016.00100 (PMC4751268; doi:10.3389/fmicb.2016.00100)
Supplement: Supplementary file 2 [file DataSheet1.pdf]

SUPPLEMENTAL INFORMATION

Sister *Dehalobacter* Genomes Reveal Specialization in Organohalide Respiration and Strain Differentiation Likely Driven by Chlorinated Substrates

Shuiquan Tang, Po-Hsiang Wang, Steven A. Higgins, Frank E. Löffler and Elizabeth A. Edwards

*** Correspondence:** Elizabeth A. Edwards elizabeth.edwards@utoronto.ca

List of Supplemental Figures

FIGURE S1. Sequence alignment of 16S rRNA genes of *Dehalobacter* sp. strain CF, UNSWDHB, PER-K23, and E1.

FIGURE S2. A maximum-likelihood phylogenetic tree of the partial genomes of five *Dehalobacter* strains.

FIGURE S3. A maximum-likelihood phylogenetic tree of all RDases (characterized or putative) from the four Dehalobacter genomes together with 15 RDases with known functions from other organisms.

FIGURE S4. Sequence alignment between strain PER-K23 and strain CF or strain E1 focused on the two *rdhA* clusters.

FIGURE S5. Pairwise, partial genome alignments of the five *Dehalobacter* strains.

FIGURE S6. *pceABCT* operon alignment of *Dehalobacter* sp. strain CF (identical to the one in strain DCA), PER-K23, and E1.

FIGURE S7. Comparison of strains CF, PER-K23 and E1 over three gene clusters related to (a) the Wood-Ljungdahl pathway, (b) molybdopterin biosynthesis, and (c) cobalamin biosynthesis.

List of Supplemental Tables

Table S1 (Separate Excel file)

TABLE S2. Comparison of transcriptional regulatory domains in the *rdhA* gene neighborhood between organohalide respiring species within the phyla *Firmicutes* and *Chloroflexi*.

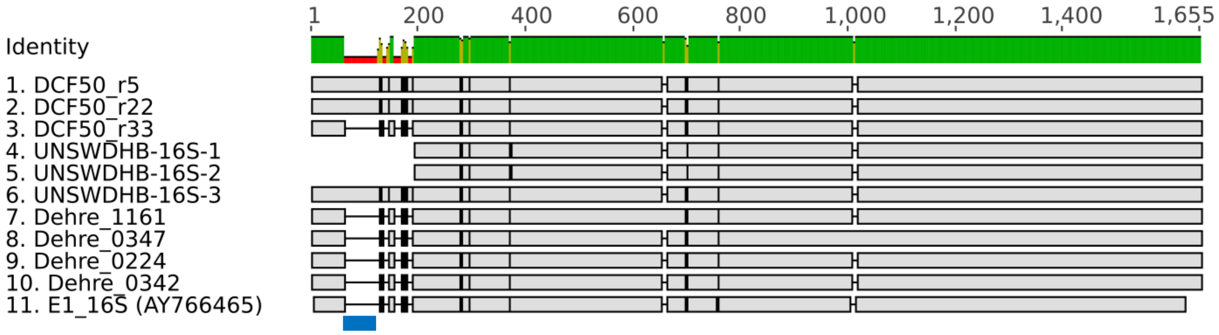

FIGURE S1. Alignment of 16S rRNA gene sequences from *Dehalobacter* sp. strain CF, UNSWDHB, PER-K23, and E1. Sequence discrepancies are highlighted in black in the aligned sequences. The first row represents the identity across all sequences for every position. Green means that the residue at the position is the same across all sequences. The blue bar (bottom left) highlights the area of the sequence insertion near the 5' end. Note the genomes of strain CF, UNSWDHB, and PER-K23 contain multiple copies of 16S rRNA genes with sequence variation.

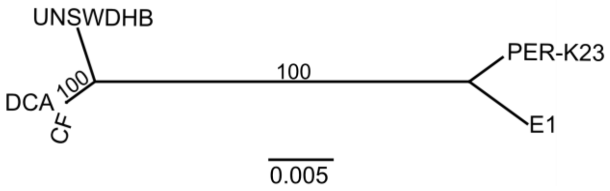

FIGURE S2. A maximum-likelihood phylogenetic tree of the partial genomes of five *Dehalobacter* strains. The tree was built based on the alignment of concatenated orthologous sequences (~ 0.9 Mb), which were derived from Mauve alignment and manual editing. Pairwise alignment of these partial genomic sequences is shown in FIGURE S7. The tree generated was using the PhyML plugin in Geneious under the substitution model of Jukes-Cantor 69 (JC69). Bootstrap support values (from 100 bootstrap iterations) are indicated where greater than 50%. The scale bar represents the average number of substitutions per site.

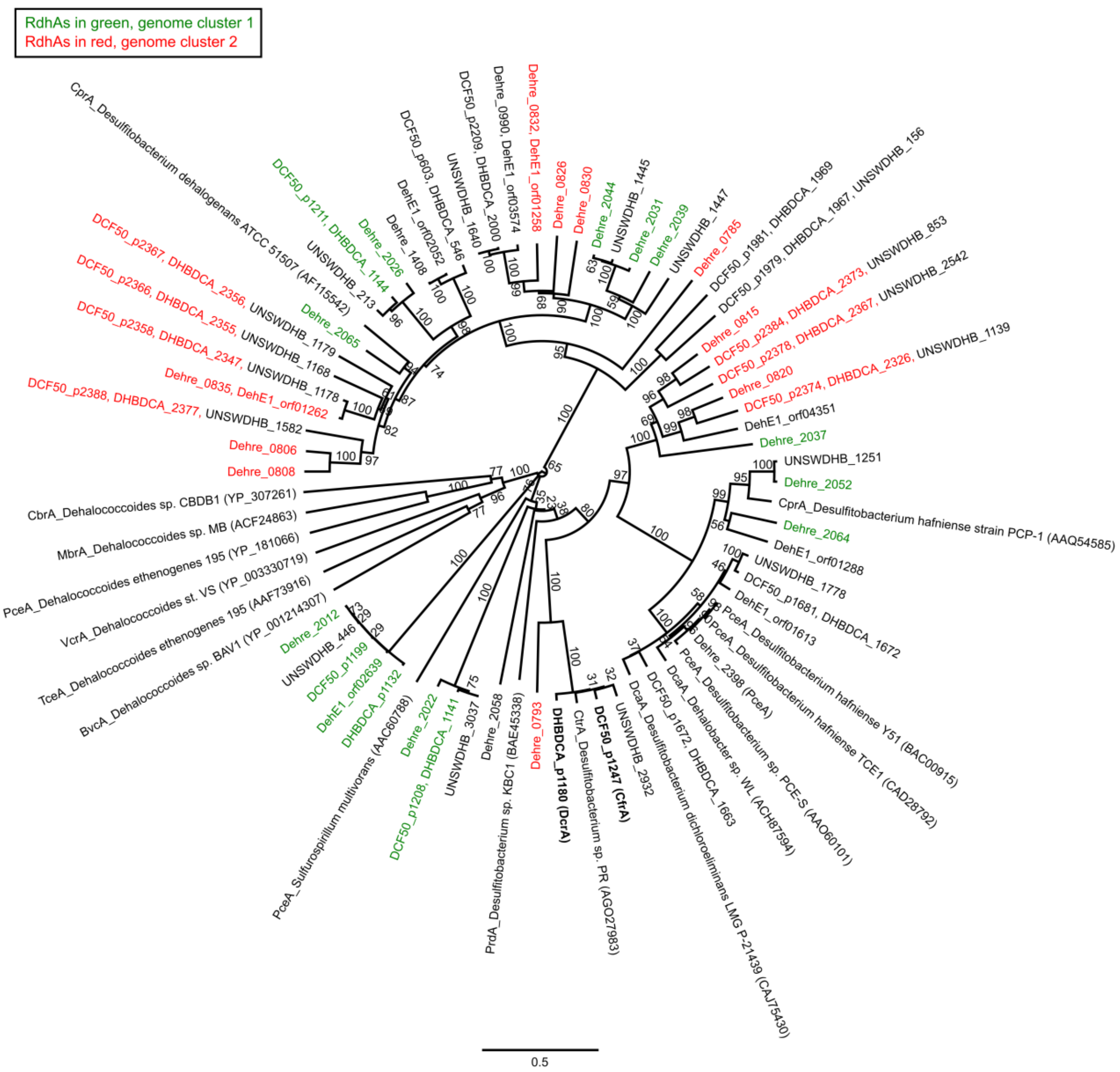

FIGURE S3. A maximum-likelihood phylogenetic tree of all RDases (characterized or putative) from the five *Dehalobacter* genomes together with 16 RDases with known functions from other organisms. The alignment was generated using the MUSCLE algorithm, and the tree generated using the PhyML plugin in Geneious under the WAG model of evolution. The scale bar represents the average number of substitutions per site. Bootstrap values from 100 iterations are shown.

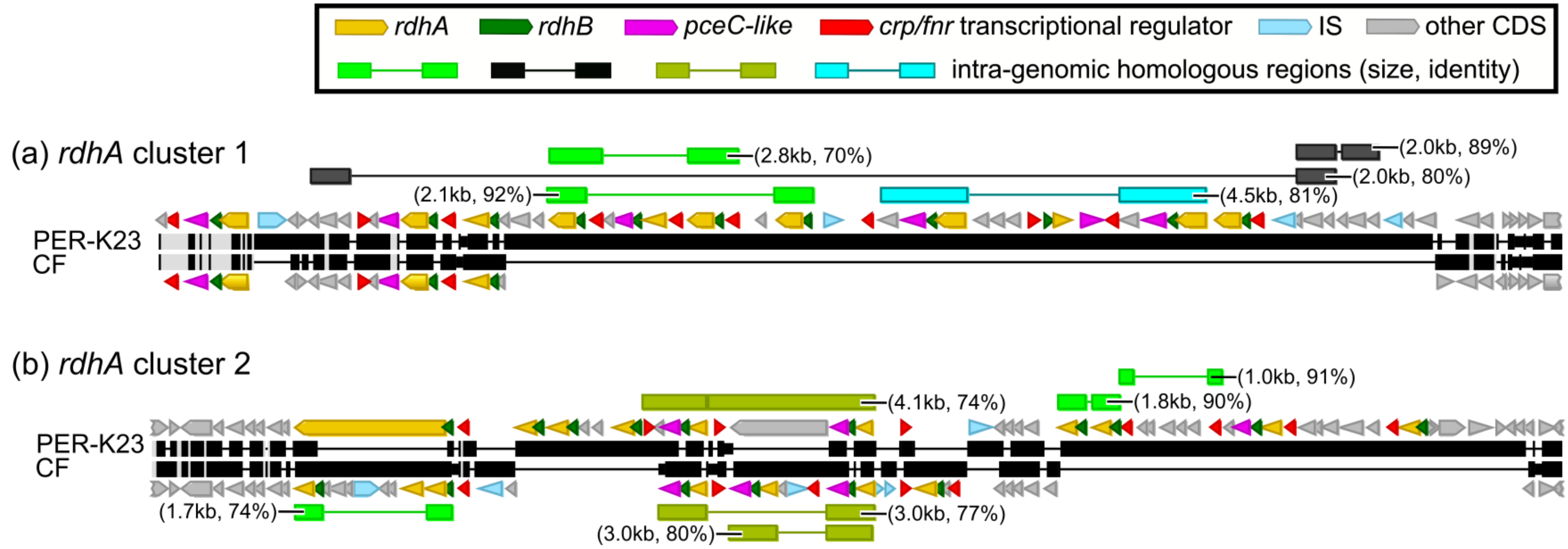

FIGURE S4. Sequence alignment of the two *rdhA* clusters comparing strains PER-K23 and CF.(a) *rdhA* cluster 1, (b) *rdhA* cluster 2. All CDSs are indicated as directional blocks in different colors: *rdhA* genes (yellow), *rdhB* genes (green), *pceC*-like genes (purple), crp/fnr transcriptional regulators (red), ISs (light blue) and others (grey). Pairwise blocks connected with lines represents intra-genomic homologous regions; size and nucleotide identity are noted in parenthesis; highly similar regions are drawn in the same color. Strain PER K23 has 11 *rdhA* genes in cluster 1 and 10 in cluster 2, while strain CF has 3 *rdhA* genes in cluster 1 and 7 in cluster 2.

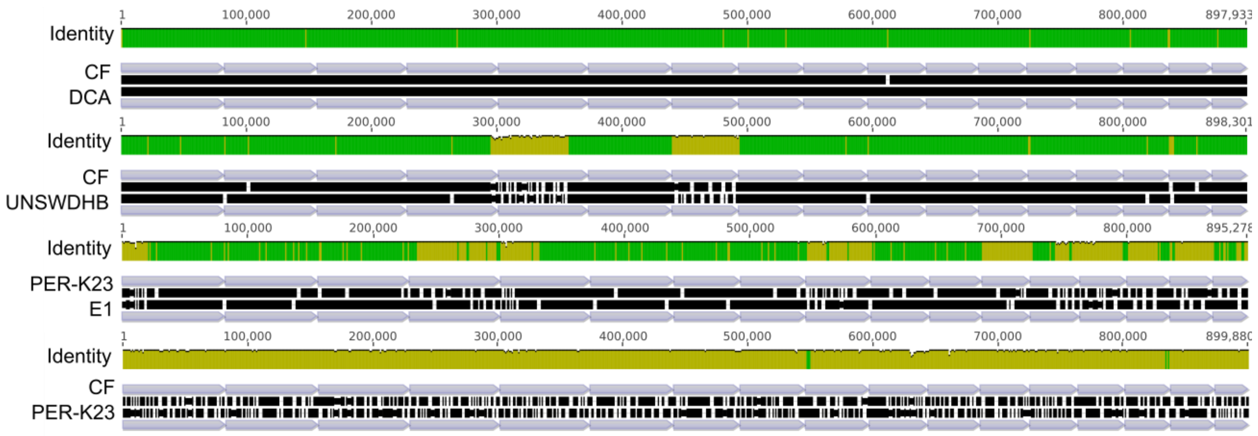

FIGURE S5. Pairwise, partial genome alignments of the five *Dehalobacter* strains. 17 large orthologous regions (arrow bars) from each genome were picked and concatenated. Sequence identity is shown on the top of each alignment, with green indicating 100% identity and yellow indicating mismatches.

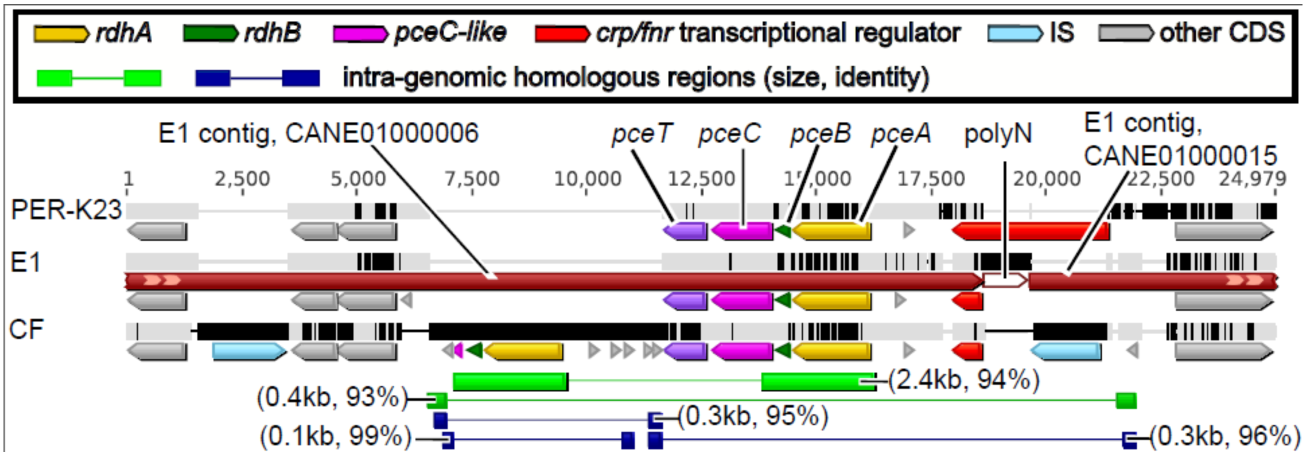

FIGURE S6. ***pceABCT*** **operon alignment of** ***Dehalobacter*** **sp. strain CF (identical to the one in strain DCA), PER-K23, and E1.** Sequences for E1 span to contigs joined by a polyN (dark red). Intra-genomic homologous regions in strain CF only are identified by a block of sequence connected by a line with size and identity between two copies in brackets. The gene landscape of this region in strain UNSWDHB is less clear due to fragmentation in the assembly of this region, but is highly similar to that of strain CF.

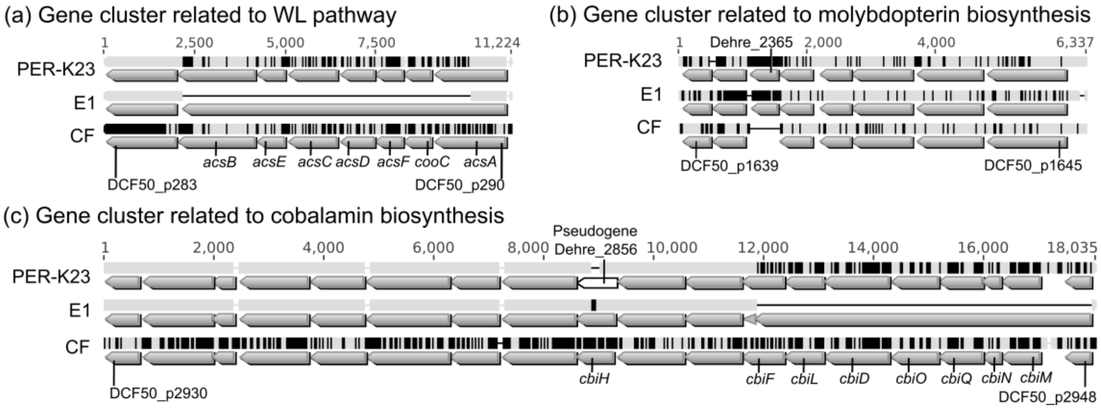

FIGURE S7. Comparison of strains CF, PER-K23 and E1 over three gene clusters related to (a) the Wood-Ljungdahl pathway, (b) molybdopterin biosynthesis, and (c) cobalamin biosynthesis. Strains CF, DCA and UNSWDHB have no significant difference in these three regions.

Table S2: Comparison of transcriptional regulatory domains in the *rdhA* gene neighborhood between organohalide respiring bacteria in the Firmicutes and Chloroflexi

				Pfam Domain (Kind, Function and Name) within 5 ORFs up or downstream from an *rdhA* gene												
				Input Small-molecule binding	Output DNA-binding	Receiver Receiver	Transmitter Histidine kinase	Transmitter Histidine kinase	Input Small-molecule binding	Input Small-molecule binding	Input Small-molecule binding	Input Small-molecule binding	Output DNA-binding	Output DNA binding	Output DNA-binding	Output DNA-binding
**Phylum**	Genus/strain	No. of *rdhA* genes used in the analysis*	No. of reported *rdhA* genes*	cNMP_binding	HTH_Crp_2	Response_reg	HisKA	HATPase_c	PAS_9	PAS_8	PAS_4	PAS	Trans_reg_C	MarR_2	HTH_21	GerE
Firmicutes	*Dhb* DCA	17	*17*	16	17	1	1	1	0	0	0	0	1	0	2	0
	Dhb CF	*17*	*17*	17	17	1	1	1	0	0	0	0	1	0	2	0
	Dhb E1	*8*	*10*	7	8	0	1	1	0	0	0	0	0	0	0	0
	Dhb PERK23	*23*	*25*	21	20	0	0	0	0	0	0	0	0	0	0	0
	Dhb UNSWDHB**	*18*	*17*	3	3	3	1	1	0	0	1	0	0	0	2	0
	Dsf DCB2	*7*	*7*	9	9	0	0	0	0	0	0	0	0	0	0	0
	Dsf dehalogenans	*7*	*7*	4	4	2	2	2	0	2	0	0	2	0	0	0
	Average per rdhA gene			0.8	0.8	0.1	0.1	0.1	0.0	0.0	0.0	0.0	0.1	0.0	0.0	0.0
Chloroflexi	*Dhc* 195	*17*	*17*	1	1	10	6	8	5	1	0	0	11	5	0	1
	Dhc BAV1	*12*	*10*	0	0	9	7	9	8	2	0	1	7	1	6	2
	Dhc BTF08	*20*	*20*	0	0	15	10	13	21	1	2	3	8	11	5	5
	Dhc CBDB1	*31*	*32*	0	0	34	27	31	36	3	4	10	26	16	1	5
	Dhc DCMB5	*23*	*23*	0	0	23	18	25	17	4	0	3	22	3	0	2
	Dhc GT	*20*	*20*	0	0	15	12	12	21	2	4	4	9	15	1	3
	Dhc VS	*36*	*36*	0	0	40	24	31	31	4	2	5	28	22	2	10
	Dhg BLDC9	20	20	0	0	6	2	5	3	4	2	0	2	0	13	5
	Average per rdhA gene			0.0	0.0	0.8	0.6	0.7	0.7	0.1	0.1	0.1	0.6	0.4	0.2	0.2

*The number of *rdhA* genes reported by the primary authors is occasionally slightly different from the number of rdhA genes identified and analyzed in this domain search.

**The incomplete draft status of the UNSWDHB may explain the relatively low numbers of regulatory domains for this organism

Shading highlights the major differences between the Firmicutes and Chloroflexi
